# Nanocrystalline Cellulose from Microcrystalline Cellulose of Date Palm Fibers as a Promising Candidate for Bio-Nanocomposites: Isolation and Characterization

**DOI:** 10.3390/ma14185313

**Published:** 2021-09-15

**Authors:** Amina Hachaichi, Benalia Kouini, Lau Kia Kian, Mohammad Asim, Hassan Fouad, Mohammad Jawaid, Mohini Sain

**Affiliations:** 1 Research Unit Materials, Processes and Environment (URMPE), Faculty of Technology, M’hamed Bougara University, Boumerdes 35000, Algeria; a.hachaichi@univ-boumerdes.dz; 2 Laboratory of Coatings, Materials and Environment, M’hamed Bougara University, Boumerdes 35000, Algeria; kouinib@gmail.com; 3 Laboratory of Biocomposite Technology, Institute of Tropical Forestry and Forest Products (INTROP), Universiti Putra Malaysia, UPM, Serdang 43400, Malaysia; laukiakian@gmail.com (L.K.K.); khanfatehvi@gmail.com (M.A.); 4 Applied Medical Science Department, Community College, King Saud University, P.O. Box 10219, Riyadh 11433, Saudi Arabia; menhfef@ksu.edu.sa; 5 Mechanical & Industrial Engineering (MIE), University of Toronto, Toronto, ON M5S 3G8, Canada; m.sain@utoronto.ca

**Keywords:** nanocrystalline cellulose, microcrystalline cellulose, date palm, morphology, crystallinity, thermal behavior

## Abstract

Date palm fiber (*Phoenix dactylifera* L.) is a natural biopolymer rich in lignocellulosic components. Its high cellulose content lends them to the extraction of tiny particles like microcrystalline cellulose (MCC) and nanocrystalline cellulose (NCC). These cellulose-derived small size particles can be used as an alternative biomaterial in wide fields of application due to their renewability and sustainability. In the present work, NCC (A) and NCC (B) were isolated from date palm MCC at 60 min and 90 min hydrolysis times, respectively. The isolated NCC product was subjected to characterization to study their properties differences. With the hydrolysis treatment, the yields of produced NCC could be attained at between 22% and 25%. The infrared-ray functional analysis also revealed the isolated NCC possessed a highly exposed cellulose compartment with minimized lignoresidues of lignin and hemicellulose. From morphology evaluation, the nanoparticles’ size was decreased gradually from NCC (A) (7.51 nm width, 139.91 nm length) to NCC (B) (4.34 nm width, 111.51 nm length) as a result of fragmentation into cellulose fibrils. The crystallinity index was found increasing from NCC (A) to NCC (B). With 90 min hydrolysis time, NCC (B) showed the highest crystallinity index of 71% due to its great cellulose rigidity. For thermal analysis, NCC (B) also exhibited stable heat resistance, in associating with its highly crystalline cellulose structure. In conclusion, the NCC isolated from date palm MCC would be a promising biomaterial for various applications such as biomedical and food packaging applications.

## 1. Introduction

Cellulose fibers are natural biopolymers found abundantly on earth and this has contributed to their great availability at low cost. They are renewable, biodegradable, biocompatible, semicrystalline, low density, and can be easily obtained from various natural fibers like roselle, date palm, bamboo, kenaf, and cotton [1,2]. Currently, the use of cellulose to develop biocomposite material has received great attention from scientific researchers due to the hierarchical structure of cellulose which allows producing microsized or nanosized dimensions chemically, mechanically or combining both means [2,3].

*Phoenix dactylifera* L., also known as the date palm tree, is a plant that belongs to the family of Arecaceae. It is broadly cultivated over the Northern Africa, Middle East, and Southern Asia regions. In Algeria, approximately 18 million date palm trees are cultivated on a 169,380 hectare area of desert. From these palm groves, over 10 million of young trees are actively harvested for an annual yield of 500,000 tons of fruit dates, with the cultivars of Ghars and Deglet Nour dates sharing up to 96% of the local market, making the country the third largest producer of dates in the world. However, the old and dead palms could generate agricultural waste, which may pose risks to environmental conservation. It is estimated that about 100,000 tons of date palm waste is generated every year in Algeria [4,5,6]. The Algerian government has an interest in supporting the using of waste as a source to develop new biomaterial to protect the environment. Therefore, the fruit bunch branches of Algerian date palm trees have become a potential raw material to be used to obtain cellulose fibers [4,5].

Although cellulose fibers have many advantages, they also have some disadvantages which limit their use in some applications, like high absorption of water, low strength, and low thermal stability. These disadvantages can be improved by converting cellulose particles to small particles, which can expand their applications [2,6]. Microcrystalline cellulose (MCC) can be extracted from disintegrated cellulose, having a diameter of 10–50 μm, and length of 100–1000 μm [7]. Their purity also makes them an ideal starting material to isolate nanocrystalline cellulose (NCC) product, which is 5–10 nm in width and 100–500 nm in length. Acidic hydrolysis is the most common method applied to remove amorphous parties in order to produce nanocrystallite cellulose with high crystallinity [2,8].

Nowadays, NCC attracts wide attention due to its extraordinary criteria like high aspect ratio, great surface area, excellent mechanical and barrier properties [9,10]. NCC can act as a reinforcing agent for nanocomposite material in diverse fields such as automobile production, aerospace, biomedical, and food packaging applications [11,12]. The variation of NCC properties largely depends on the origin of fibers as well as the treatment process, including the effect of parameters like acid concentration, temperature, and reaction time [13,14,15]. Foo et al. [16] successfully tailored the properties of NCC with attained optimal yield and crystallinity, while at the lowest particle size for large-scale production by using a lower sulfuric acid amount than the common hydrolysis condition. Qian et al. [17] produced high yield of NCC by employing pretreatment of microwave-integrated acid hydrolysis in combination with an enzymatic reaction process. Another study by Tuerxun et al. [18] had conducted a series of mechanical-chemical purification steps, comprising hydrogen peroxide bleaching, alkali treatment, and acid hydrolysis to isolate NCC from rubberwood and kenaf fibers. Furthermore, Ogundare et al. [19] had obtained cigarette-filters-derived NCC via ethanol extraction, chlorite bleaching, alkaline deacetylation, and ultimately followed by sulfuric acid hydrolysis. Additionally, Liu et al. [20] extracted NCC from industrial kelp waste by utilizing a 51 wt% sulfuric acid concentration, while Dunlop et al. [21] isolated NCC from tunicates using a prehydrolysis-kraft cooking-bleaching method. Therefore, a series of well-designed chemical treatments involving the acid hydrolysis reaction is crucial for the preparation of NCC.

In the present work, the isolated MCC from date palm fiber in our previous work [4] was utilized as the starting material for NCC isolation by using an acid hydrolysis process, with different reaction times, in the aim to examine the influence of acidic hydrolysis time on NCC properties. Meanwhile, the NCC characterization was implemented through field-emission scanning electron microscopy (FESEM), transmission electron microscopy (TEM), energy dispersive X-Ray (EDX), Fourier transform infrared rays (FTIR), thermogravimetric analysis (TGA), differential scanning calorimetry (DSC), and zeta potential, to assess the feasibility of isolated NCC from date palm MCC and its potential use as a reinforcing component in the bio-nanocomposites field.

## 2. Materials and Methods

### 2.1. Materials

Date palm MCC (MCC-DP) was produced as reported in our previous article by using the fruit bunch branch fibers part [4]. Sulfuric acid (purity: 95–97%) and membrane dialysis tubing (average flat width: 10 mm; molecular weight cut-off: 2000) were procured by the Sigma-Aldrich Company (St. Louis, MI, USA).

### 2.2. Isolation Method of NCC

MCC-DP was utilized as starting biomass to extract NCC product. Firstly, a 10 g MCC-DP was treated by 64 wt% sulfuric acid at 60 °C under strong agitation, separately in two different reaction times of 60 and 90 min. At the end of reaction time, about 800 mL of ice-cold distilled water was poured into the solution to quench the acid reaction. After that, the hydrolyzed suspension underwent centrifugation with 1612.8× *g* at 10 °C for 10 min to neutralize the NCC suspension until pH 3 was achieved (4 to 5 water cycles). Afterwards, the suspension was dialyzed by using dialysis membrane tubing for 7 days to further increase its pH to 5, followed by the ultrasonication (Sonic Vibra-Cell, VCX 500, Newton CT, USA) treatment for 30 min (with settings: 6 s on/2 s off; 20 kHz frequency; 40% amplitude; 500 W power output) to form a homogeneously dispersed white colloidal suspension. Ultimately, the supernatant portion underwent a freeze-drying (Labconco FreeZone, 230 V, Kansas, MO, USA) process at for 4 days (with settings: 50 Pa vacuum pressure; −20 °C temperature of lyophilization) to form the NCC powder product.

### 2.3. Characterization Methods

#### 2.3.1. Yield Determination

The NCC yield was determined using Equation (1), as given:

(1) $$\mathrm{Yield}\;\left(\%\right)=\frac{M1}{M2}\times 100\%$$

in which M1 is the weight of oven-dried NCC, and M2 is the weight of MCC-DP.

#### 2.3.2. FTIR Examination

The surface functionality for freeze-dried NCC samples was investigated by using an infrared-ray spectrophotometer (Perkin Elmer 1600, Ramsey, MN, USA) in 650–4000 cm^−1^ wavenumber range with 32 total scans at 4 cm^−1^ resolution. A software of Nicolet OMNIC 5.01 was applied to determine the prominent transmittance peaks at specific points.

#### 2.3.3. Structure, Elemental Composition, Particle Size Distribution and Zeta Potential Analysis

To identify the structure and elemental composition of the NCCs, a FESEM microscope (JEOL JSM-7000F, Akishima, Tokyo, Japan) with EDX was used under accelerated voltage of 10–20 kV. Before viewing, the NCC powders were evenly distributed by sticking on the aluminum stubs that were mounted with carbon tapes and then sprayed with platinum coating to avoid charging. Besides this, the nanostructure of NCC samples was examined by using a TEM microscope (FEI Tecnai G2 F30, Atlanta, GA, USA), under an accelerating voltage of 200 kV. Before viewing, a 2% uranyl acetate solution was used to stain the negatively charged NCC liquid samples prior to deposition on copper grid substrate. The nanoparticle sizes of NCC were determined using ImageJ software (LOCI, University of Wisconsin: Madison, WI, USA). In addition, the zeta potential of NCC suspension was examined by using a Malvern Zeta sizer Nano ZS90) equipment, Malvern Panalytical B.V., Brighton, UK (with 12 repeated runs at 2 mm measurement to obtain average values.

#### 2.3.4. Crystallinity Analysis

The crystallinity degree for each NCC was assessed using a X-ray diffractometer (SHIMADZU XRD-6000, Kyoto, Japan) with Cu Kα radiation ranging from 5° to 60° at 2 °C/min stepwise scanning, which operating at 30 kV voltage and 30 mA current. Crystallinity index (CrI) was determined following Equation (2) [22]:

(2) $$\mathrm{CrI}\;\left(\%\right)=(I_{200}-I_{\mathrm{am}})/I_{200}\times 100\%$$

in which, I_200_: maximum intensity point at 22.5° corresponding to the crystalline plateau; I_am_: minimum intensity point at 16.3° corresponding to the crystalline background.

#### 2.3.5. Thermal Analysis

About 6 mg for each NCC sample was analyzed for their thermal stability using a TA-SDT Q600 thermo gravimetric analyzer (Mettler-Toledo International Inc., Columbus, OH, USA) under N_2_ gas atmosphere at 10 °C/min heating rate with 30–650 °C temperature range. Also, a Differential Scanning Calorimetry (DSC) (Mettler-Toledo International Inc., Columbus, OH, USA) was used to evaluate the alteration of NCC thermal molecules behavior. The samples were run from 30 °C to 250 °C at heating rate of 20 °C/min within a N_2_ atmosphere.

## 3. Results and Discussion

### 3.1. The Yield and Infrared Spectroscopy

In this work, the hydrolysis reaction times of 60 and 90 min were selected since they can provide optimized conditions in producing nanosized NCC particles. The designation for NCCs product with different hydrolysis times and their yields are presented in Table 1. The results show that NCC (B) reached the highest yield of 25% with an increase of 3% compared to NCC (A) of 22%, and this implied the gradual disintegration of microcellulose into crystalline nanocellulose [2]. In this work, the obtained yield of 25% for NCC (B) sample was greater than the reported studies by Kian et al. [13], who using 35 wt% concentrated sulfuric acid to yield 17.5%, 18.9%, and 16.4% NCC product at 30 min, 45 min, and 60 min hydrolysis times respectively. Additionally, the yield for the NCC (A) sample was also comparable to the yield obtained by Rasheed et al. [1], who applied the same concentration of sulfuric acid (64 wt%) at 45 min.

**Figure 1:**
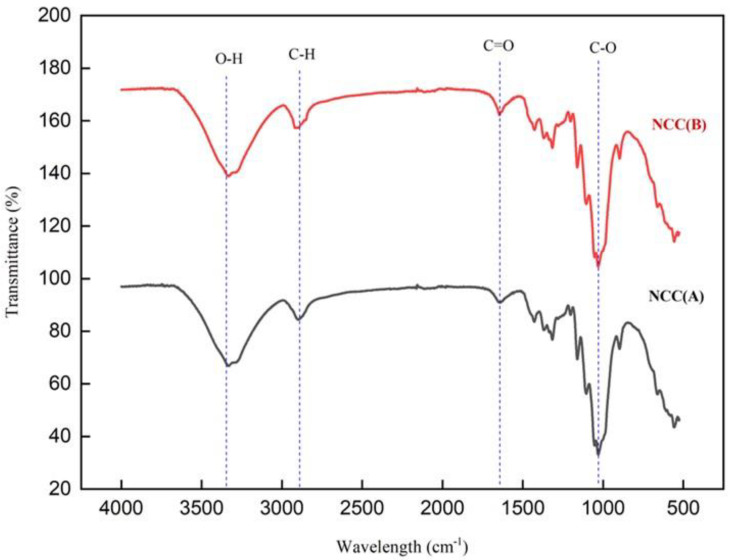
FTIR spectrum of NCC (A) and NCC (B).

The infrared spectra of NCC (A) and NCC (B) are displayed in Figure 1. Both samples revealed the same pattern of diffractions spectra, indicating the employment of different hydrolysis times in the extraction of NCC did not alter the surface functional groups of NCCs. A wide absorbed band was observed between 3300 cm^−1^ and 3500 cm^−1^, which related to O-H vibration bonds [23,24]. This absorbance becomes more intense with the extension of time from 60 to 90 min, revealing the reflectance of hydrophilic properties [25]. Another absorption peak at 2900 cm^−1^ was assigned to the C-H symmetric groups of cellulose, which was more visible after 90 min of reaction [24,25]. Meanwhile, an intense peak appeared at 1645 cm^−1^, correlating with the water-cellulose interaction chemical group [26]. Furthermore, two peaks at 1427 cm^−1^ and 1332 cm^−1^ were attributed to the C-H bonds of cellulose. The intensity of these peaks changed slightly between NCC (A) and NCC (B), a result of crystal lattice reorientation [27]. Additionally, the absorbance peaks observed at 1104 cm^−1^ correlated with C-O vibrational bonds, while the peak at 1159 cm^−1^ correlated with cellulose glycoside bonds (C-O-C) [28]. A sharp peak at 1058 cm^−1^ in relation to vibrational (C-O-C) pyranose ring of cellulose was observed for both NCC samples, proving the uninterrupted internal cellulose structure [29]. Moreover, the characteristic peak at 897 cm^−1^, in associating to C-OS vibrational bonds of C-O-SO^3−^ groups, appeared in both NCC (A) and NCC (B) samples. This evidenced the sulfation reaction had successfully occurred on cellulose surface via sulfuric acid hydrolysis [30]. As an interesting remark, we noticed that the hemicellulose traces in relating to peak at around 1740 cm^−1^, were completely absent, suggesting the purity of produced NCCs in this work when comparing to our previous study for MCC-DP [4].

### 3.2. Morphology, Particle Size, Element Composition, and Zeta Potential Analysis

**Figure 2:**
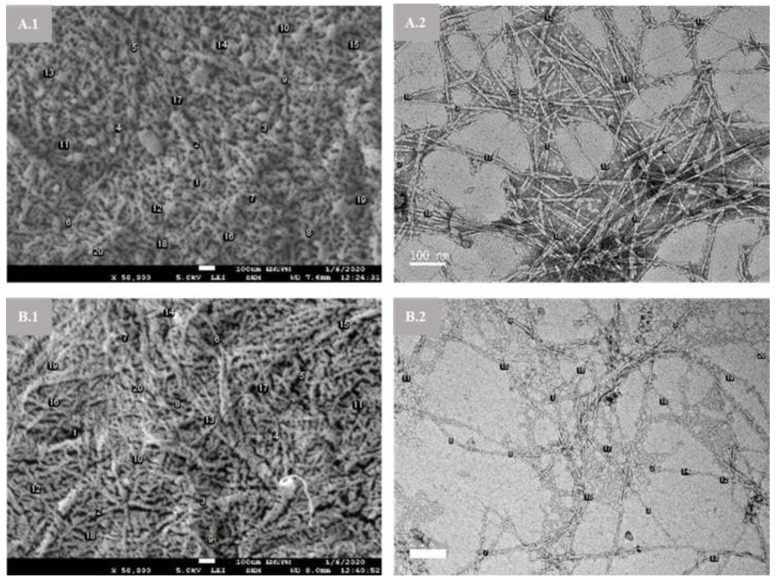
FESEM (**A.1**,**B.1**) and TEM (**A.2**,**B.2**) images of NCC (**A**) and NCC (**B**) samples.

Morphology examination for NCC (A) and NCC (B) samples by FESEM and TEM are illustrated in Figure 2. In Figure 2A, NCC (A) showed needle-like particles due to the disintegration MCC-DP amorphous part by hydronium ions attack and then resulted in released cellulose nanocrystals [31]. The extended hydrolysis time from 60 to 90 min promoted more isolated individual crystallites as presented by NCC (B) in Figure 2B. This was owing to further weakened hydrogen bonding between cellulose molecular chains [32]. In addition, both NCC (A) and NCC (B) showed homogeneous dispersion behavior, which related to the good stability of the colloidal suspension [29]. Meanwhile, the size of nanoparticles was measured and presented in Table 2. NCC (A) exhibited larger sizes in width and length compared to NCC (B), due to the gradual disintegration of NCC (A) after prolonged hydrolysis time [33]. Moreover, the features of both nanoparticle samples in the presented work are very interesting with their elongated shape of nanostructures that imparted them with high aspect ratio (diameter/length), which is better than reported in NCC studies on bamboo [1], olive [13], kenaf [34], jute [35] and sisal [36] fibers. Many studies suggested the high aspect ratio can give a strong stiffness to the percolating nanoparticle network and thus achieve high reinforcing effect in polymer matrices [37].

**Figure 3:**
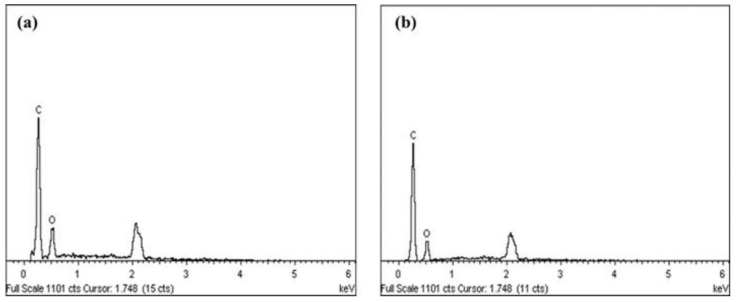
EDX spectra of NCC (A) (**a**) and NCC (B) (**b**).

EDX spectra of NCC samples showed the peaks of carbon and oxygen elements, representing a classic cellulose composition (Figure 3). The obtained results confirmed that the prolonged hydrolysis time didn’t change the elemental composition of nanoparticles, supporting the statement as analyzed by FTIR results. However, a slight difference was observed in the percentages for carbon and oxygen, which changed from NCC (A) with 78.58% carbon and 21.42% oxygen to NCC (B) with 82.07% carbon and 17.99% oxygen, probably resulting from the removal of amorphous residues [2,38].

Zeta potential is a useful analysis technique employed to examine the stability of NCCs’ dispersion in aqueous solution, which is studied via the electrostatic repulsive interaction between particles with reduced coagulation or flocculation [39]. Zeta potential of NCC (A) and NCC (B) in neutral water showed a negative charge density [23] (Table 2), owing to sulfate esters groups (–OSO^3−^) presented on NCC surface following acid hydrolysis reaction of cellulose using sulfuric acid (H_2_SO_4_). The negative values of both NCC (A) and NCC (B) were greater than −15 mV, indicating the stable dispersion of nanoparticles in aqueous solution. It could help to enhance its distribution in polymer matrix during the composite fabrication process [14,26].

### 3.3. X-ray Diffraction (XRD)

**Figure 4:**
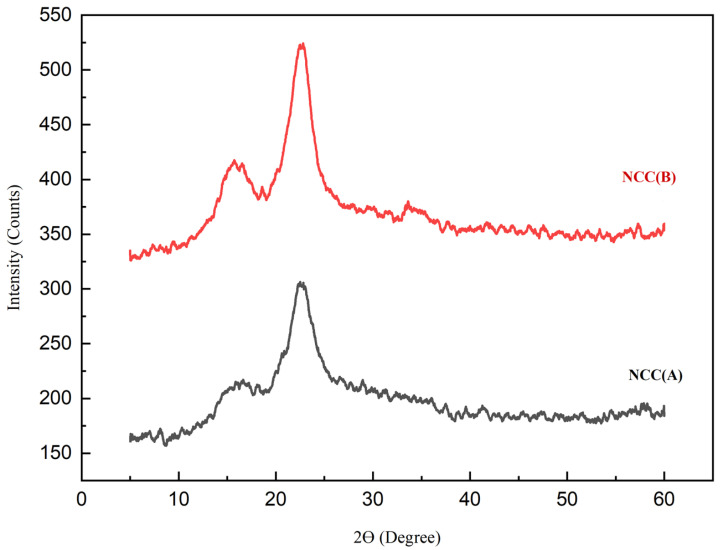
XRD plots of NCC (A) and NCC (B).

X-ray diffractograms of NCC (A) and NCC (B) are displayed in Figure 4. The main diffraction peaks appeared for both nanocelluloses at around 15.3°, 16.7°, and 22.5°, which relate to the crystallography planes of (1–10), (110), and (200), respectively. This reflected the Iβ type of cellulose structure existing within the nanocellulose structure [40,41]. In comparison, the NCC (A) sample exhibited an insignificant peak at 34.2°, with reduced intensity at both the 15.3° and 16.7° peaks, likely attributing to its less crystalline cellulose structure promoted by short hydrolysis time. Meanwhile, the diffraction peak at 22.5° demonstrated a narrower and sharper peak for NCC (B) when compared to NCC (A), which related to the reordered crystalline arrangement owing to the enhanced inter- and intramolecular hydrogen bonding [42]. With the highly intense peaks, NCC (B) presented greater CrI of 71% as compared to NCC (A) with 68% crystallinity. It was because the prolonged hydrolysis time induced the release of NCC particles from MCC-DP [13]. The crystallinity obtained in this study (71%) was comparable with the reported studies by Sung et al. [43], who isolated NCC from coffee silver skin with 72% crystallinity, and Espino et al. [44] who produced NCC from barley and Agave tequilana with crystallinity of 71%. Furthermore, it was also higher than the NCC extracted from corrugated old container fiber by Tang et al. [45] with 57.8% crystallinity.

### 3.4. Thermal Properties

**Figure 5:**
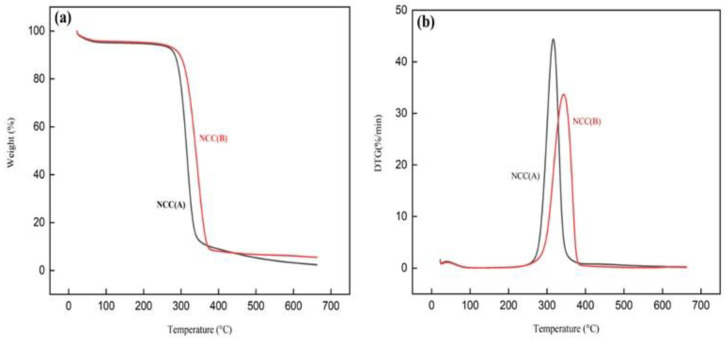
(**a**) TGA and (**b**) DTG patterns of NCC (A) and NCC (B) samples.

TGA and derivative thermogravimetric (DTG) patterns of NCCs were presented in Figure 5, while the thermal analysis data were listed in Table 3. At the beginning (Figure 5a), each sample lost weight in the temperature range of 30–140 °C, due to the evaporation of volatile components and residual water [46,47]. Above 200 °C, the NCC (B) presented a greater initial decomposition temperature at 312.23 °C, by attributing to its more crystalline structure compared to NCC (A) [47,48]. From Figure 5b, NCC (B) displayed a higher peak temperature (T_peak_), signaling the thermally stable cellulose compartment. This was possibly the existence of amorphous regions in NCC (A) that decreased their T_peak_ value. Furthermore, the sharpness of peak degradation for NCC (A) was narrower compared to NCC (B), signaling the purity of cellulose in the NCC (A) sample [30]. Beyond 400 °C, the biomass weight had reduced for both NCC (A) and NCC (B). However, NCC (B) showed a higher char residue weight of 5.49% compared to NCC (A) with 2.40%. It could be associated with the flame-retardant behavior of the highly crystalline NCC (B) structure [46,49]. Similar results were reported for NCC extracted from different natural fibers such as roselle, bamboo, and olive fiber [1,2,13]. Furthermore, the great weight losses exceeding 90% demonstrated that the structural organization of cellulose was relatively consistent, making it an ideal material for reinforcement application in high-temperature condition [13].

**Figure 6:**
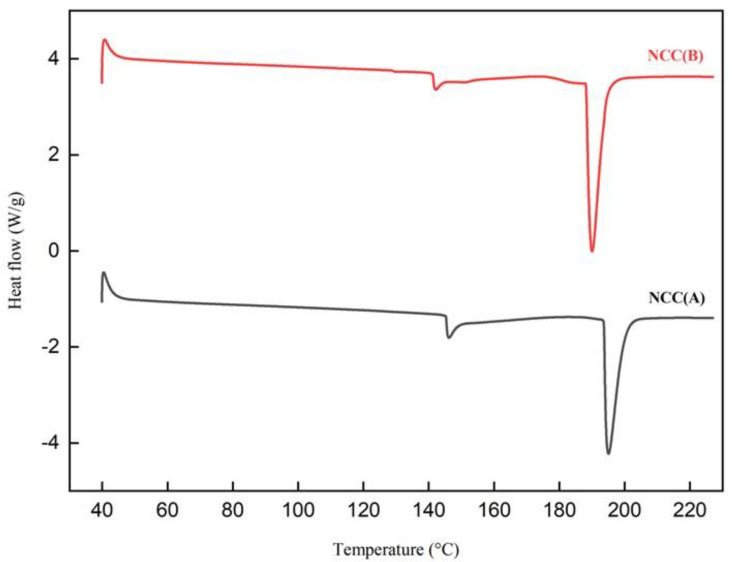
DSC patterns of NCC (A) and NCC (B) samples.

The DSC patterns of NCCs are displayed in Figure 6. From the curves, only two major endothermic peaks were observed, implying relatively stable thermal changing behavior of the isolated nanoparticles. The first endothermic peaks occurred in the 140–150 °C region, associated with the vaporization of remaining water absorbed by cellulose. At this stage, heat energy was absorbed to initiate vaporization [50,51]. Meanwhile, in the 180–200 °C region, the second endothermic peaks appeared for NCC (A) at 195.06 °C and NCC (B) at 189.34 °C. These endotherms were correlated with the breakdown of the Van der Waals interaction between cellulose hydroxyl groups that required high heat energy absorption. This behavior of heat absorption subsequently resulted in decarboxylation and depolymerization of cellulose. This was in agreement with the TGA analysis, in which the decomposition of cellulose had started to prevail at the temperatures of 293.94 °C and 312.23 °C for NCC (A) and NCC (B), respectively [27].

## 4. Conclusions

From the overall results in this work, the following major findings can be concluded. The isolation of NCC from MCC-DP was conducted successfully by sulfuric acid hydrolysis, in which the acidic concentration (64 wt%) and temperature (60 °C) were maintained constantly at varying hydrolysis reaction times (60 and 90 min). FTIR analysis showed there is no influence on cellulose surface functionality with prolonged hydrolysis reaction. Furthermore, the yield of nanoparticles increases from NCC (A) with 22% to NCC (B) with 25%. From morphological analysis by FESEM and TEM, the samples of NCC (A) and NCC (B) exhibited visible needles which indicated the successful extraction of NCC from MCC-DP at both hydrolysis times of 60 and 90 min. The analysis of XRD spectra had proven the NCC (B) with 90 min exhibited a high CrI of 71%, which qualified its use in high mechanical applications. Moreover, in thermal analysis, NCC (B) had the greatest thermostability, which suggests it could be used in high-temperature fabrication processes. Hence, the NCC isolated in this work can be utilized as a potential biofiller for manufacturing nanocomposite products in the future.

## Figures and Tables

**Table 1:** Designation of NCC products and their yields at different hydrolysis times.

Designation of NCC Product	Hydrolysis Reaction Times (min)	Yield (%)
NCC (A)	60	22
NCC (B)	90	25

**Table 2:** Size of particle, elements and zeta potential analysis of NCC (A) and NCC (B).

Samples	Width Size Average (nm)	Length Size Average (nm)	Carbon (%)	Oxygen (%)	Zeta Potential (mV)
NCC (A)	7.51	139.91	78.58	21.42	−33.7
NCC (B)	4.34	111.51	82.01	17.99	−27.9

**Table 3:** Thermal data of NCC (A) and NCC (B).

Samples	TGA Analysis				DSC Analysis		
	T_initial_ (°C) ^a^	T_peak_ (°C) ^b^	W_loss_ (%) ^c^	W_residue_ (%) ^d^	T_initial_ (°C) ^e^	T_peak_ (°C) ^f^	ΔH (J/g) ^g^
NCC (A)	293.94	315.9	92.56	2.40	193.67	195.06	70.5
NCC (B)	312.23	344.4	90.08	5.49	188.20	189.34	82.29

^a^ Initial decomposition temperature (TGA); ^b^ peak temperature on maximum lost weight (DTG); ^c^ maximum lost weight (TGA); ^d^ residual char weight (TGA); ^e^ initial degradation temperature (DSC); ^f^ peak temperature (DSC); ^g^ enthalpy change (DSC).

## Data Availability

Not applicable.
